# Parallel processing of cognitive and physical demands in left and right prefrontal cortices during smartphone use while walking

**DOI:** 10.1186/s12868-016-0244-0

**Published:** 2016-02-01

**Authors:** Naoyuki Takeuchi, Takayuki Mori, Yoshimi Suzukamo, Naofumi Tanaka, Shin-Ichi Izumi

**Affiliations:** Department of Physical Medicine and Rehabilitation, Tohoku University Graduate School of Medicine, 2-1 Seiryo-cho, Aoba-ku, Sendai, 980-8575 Japan

**Keywords:** Smartphone, Dual-task, Near-infrared spectroscopy, Prefrontal cortex, Prioritization

## Abstract

**Background:**

Smartphone use while walking is becoming a public concern owing to an increased risk of falling that can result from cognitive-motor interference. We evaluated prefrontal cortex (PFC) activity in participants playing a smartphone game while walking, in order to elucidate the role of the PFC in the allocation of attention between physical and cognitive demands. Sixteen young and 15 older adults participated in this study. Participants were instructed to perform a touch number-selecting game on a smartphone while walking. The numbers of correct and mistake responses were analyzed as a measure of cognitive performance. Linear trunk accelerations were measured by another smartphone and analyzed for step time and acceleration magnitude as an assay of gait performance. PFC activity during the task was measured using a wearable 16-channel near-infrared spectroscopy system.

**Results:**

Smartphone game playing while walking decreased the cognitive and gait performances compared with performances of single-task condition in older group more than in young group. There was no difference in PFC activation during smartphone use while walking between young and older groups, but age appeared to mediate correlation magnitude between PFC activation and changes in performance. In young adults, multiple regression analysis revealed an association of the right PFC with a reduction in acceleration magnitude (β = 0.581, *p* = 0.023), and an association of the left PFC with an increase in game-playing mistakes (β = −0.556, *p* = 0.032) during smartphone use while walking. In older adults, multiple regression analysis revealed an association of the middle PFC with a prolongation of step time (β = −0.550, *p* = 0.042) and of the left PFC with a reduction in acceleration magnitude (β = −0.648, *p* = 0.012).

**Conclusion:**

In young adults, the left PFC inhibited inappropriate action and the right PFC stabilized gait performance. In older adults, a less-lateralized PFC activity pattern suppressed the deterioration of gait performance, but this resulted in impairment on a simultaneous cognitive task. These results suggest that lateralization of motor and cognitive tasks aids in efficient task completion during a complex action such as using a smartphone while walking.

## Background

Smartphones are rapidly becoming prevalent in modern society and their use while walking is increasing in daily life. Smartphone use while walking is regarded as a type of dual task, which requires an appropriate allocation of cognitive and physical resources to each task [1]. Overload of central resources is associated with an inability to allocate attention appropriately between simultaneously performed cognitive and physical tasks. Therefore, smartphone use while walking is becoming a public concern with respect to the risk of collisions and falls, due to cognitive-motor interference as well as reduced visual information of surroundings [2–4].

In general, dual tasks are thought to be destabilizing because they involve competing demands for cognitive and physical resources; this effect is termed dual-task cost, wherein cognitive-motor interference can cause deterioration of one or both tasks [5, 6]. Dual-task cost can be observed more consistently in older adults, in whom it is commonly reported that dual-task walking reduced gait speed and cognitive performance. This deterioration of both cognitive and physical performance in the dual-task condition is considered to result from prioritization of gait stability over the cognitive task to compensate for a lower postural control ability in older adults, a phenomenon termed the “posture-first strategy” [7, 8]. Moreover, effective prioritization of simultaneously performed tasks can be impaired, resulting in fall risk, when cognitive flexibility is limited [8, 9]. In contrast, it has been reported that sufficient postural control ability and self-awareness allow young healthy participants initially to allocate more attention toward the cognitive task than toward gait stability [8].

Many studies have reported that dual tasks activate the prefrontal cortex (PFC), which plays roles in executive functions such as attention and multi-tasking (for a review, see [10]). Studies have found that dual tasks, such as verbal fluency or calculating during walking, activate the PFC [11–13]. Although to our knowledge, there are no reports of PFC activation during cellphone or smartphone use while walking, findings about other dual tasks indicate that smartphone use while walking may also activate the PFC owing to increased demand on executive functions. Moreover, among dual tasks, smartphone use while walking is unique, in that it decreases the availability of visual information about surroundings and modifies physical demands associated with manipulation of the smartphone itself. Therefore, the model of smartphone use while walking provides an opportunity to determine the effects of cognitive-motor interference on dual task performance [4].

Dual-task cost has been correlated with cognitive function, in particular, attention and executive function [5, 9]. Considering that PFC function is important for executive functions, including attention, selection, and monitoring (for reviews, see [14, 15]), PFC activation might be related to the allocation of attention between cognitive and physical performances during dual tasks. However, it remains unknown how the PFC contributes to cognitive and physical demands during dual tasks. Moreover, in addition to postural control ability, the change in PFC function might influence cognitive and physical dual-task costs in an age-dependent manner, as the PFC is highly susceptible to age-associated changes and its functions decline early with aging [16–18]. Recently, it has been reported that PFC activation during dual tasks differs between younger and older participants [13, 19], but it remains to be clarified how these age-dependent changes in PFC function influence changes in dual-task cost at different ages.

In this study, by using a wearable near-infrared spectroscopy (NIRS) system, we evaluated PFC activity during a dual task consisting of smartphone game playing while walking on the floor. We hypothesized that the PFC plays an important role in allocation of attention between cognitive and physical demands during smartphone use while walking. Moreover, we hypothesized that the influence of PFC activation on cognitive and physical demands differs depending on subjects’ age. In order to investigate these hypotheses, we evaluated the correlation between PFC activity and dual-task cost during smartphone use while walking in both young and older subjects. Understanding the role of PFC function in allocation of attention between cognitive and physical demands might elucidate the severity of the risk of falling due to smartphone use while walking.

## Methods

### Participants

Sixteen young adults (“young group,” 11 men and 5 women, mean age 25.9 ± 4.4 years, range 20–33) and 15 older adults (“older group,” 10 men and 5 women, mean age 71.7 ± 3.3 years, range 65–78) participated in this study. Mini-Mental State Examination (MMSE) performances [20] were well above the suggested dementia cutoff score of 24 in all participants. All were right-handed and had no neurological abnormalities. All participants gave written, informed consent, and the protocol used in the study was approved by the local ethics committee of the Tohoku University Graduate School of Medicine (reference no. 2013-1-511).

### Smartphone task

**Fig. 1:**
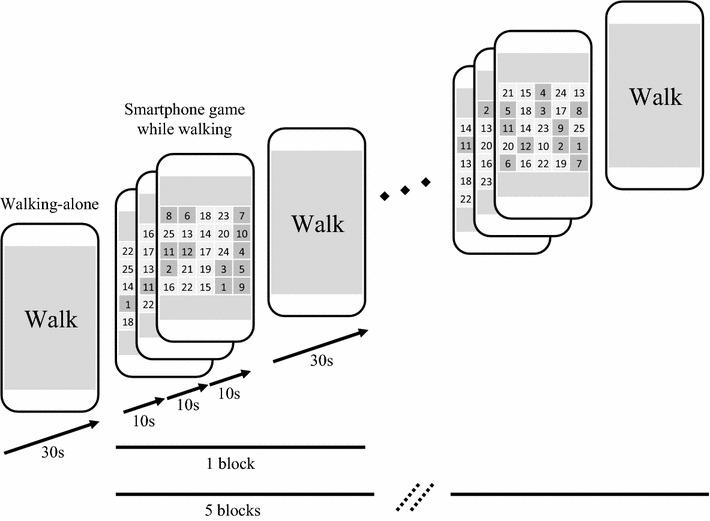
Flow of smartphone task. Participants are instructed to touch in ascending order numbers placed randomly in a 5 × 5 matrix on the smartphone screen, while walking at a comfortable speed. A new set is presented every 10 s and three sets are presented in the task condition. The *color* of the matrix cell changes when the number is unambiguously touched. The control condition is walking only. The session begins with the control condition, walking-only for 30 s, followed by five experimental blocks, each consisting of three 10-s replicates of the task followed by the walking-only condition

Participants were instructed to perform a number-selecting task on a smartphone while walking at a comfortable pace on the floor. A treadmill was not used because we anticipated that a change in gait pattern would be more clearly detected on the floor at an unregulated speed than on a treadmill at a constant speed [21]. The smartphones used in this study (dimensions, mm: 58.6 width, 123.4 height, 6.1 depth; weight: 88 g; iPod Touch 5; iOS 7.1.1; Apple Inc., USA) included a 3-axis acceleration sensor, a recording device, and a computer program for processing the acceleration signals. We modified and used the free game *Touch the Numbers* (Tekunodo Inc., Japan). Participants were instructed to touch in ascending order each number of a set of numbers distributed randomly over a 5 × 5 matrix on the smartphone screen; a new set was presented every 10 s, and three sets were presented in the task condition (Fig. 1). The session began with the control condition, walking-only for 30 s, followed by five experimental blocks, each consisting of three 10-s replicates of the task followed by 30 s of walking only. As a result, participants completed the smartphone task a total of 15 times per dual task session. The numbers of correct and mistake responses were automatically recorded by the smartphone. Participants were instructed to walk continuously around a circle about 2.5 m in radius. We selected the physical task of walking around a circle in order to avoid the extra movement that would accompany a turn and to provide constant walking condition across the floor in the limited space of the experimental room for the duration of the experiment. The participants were instructed to keep their faces turned to the screen of the smartphone and to minimize head movements in all conditions. This instruction was aimed to avoid a reduction of gait change by visual compensation and to eliminate the possibility that attention to the smartphone screen may drive the difference in PFC activity between walking alone and smartphone use while walking. All participants were instructed to wear their usual walking shoes and to avoid high heels and hard-soled shoes. The participants were allowed to familiarize themselves sufficiently with the number-touching task before measurements began. Participants were asked not to consciously prioritize either task over the other, in order to minimize task self-prioritization effects [22, 23]. Participants also performed the touch game while sitting as same as dual task condition. This sitting condition was designed to enable comparison of smartphone game performance between single and dual task conditions. The order of sitting and walking conditions was counterbalanced. The means of correct and mistake responses in each condition were used for statistical comparisons.

### Gait parameters

In order to evaluate gait performance, trunk linear accelerations were measured using the built-in acceleration sensor of another iPod device as described previously [24]. It was attached to the L3 spinous process using a semi-elastic belt. Acceleration data were recorded with a sampling frequency of 50 Hz using an application developed in the iOS environment. We used a time-adjustment application (Jikoku-tyousei Assistant, made by Takeshi Yasukawa) to synchronize the timing between the game task and the acceleration-measuring iPod devices. In off-line analysis, acceleration data for each 30-s task were filtered with a low pass 4th order bi-directional Butterworth filter at 10 Hz. To account for variations in iPod orientation, the total mean acceleration was subtracted from the acceleration data. We calculated two gait parameters, step time and acceleration magnitude, using trunk acceleration, as described previously [24–26]. Step time was obtained by calculating the interval time between acceleration peaks. Acceleration peaks were automatically detected by the *findpeaks* function in MATLAB (MathWorks, Natick MA) custom scripts using a threshold of one standard deviation to determine if each peak was significantly higher than the data around it. Peak-to-peak intervals were excluded from analysis if time to complete a cycle was <200 ms. Acceleration magnitudes were obtained by calculating the root mean square of acceleration data [25]. For statistical analysis, we averaged step time and acceleration magnitude values across those calculated for the antero-posterior and vertical directions, because the medio-lateral direction shows a monophasic pattern over the course of one stride, whereas the antero-posterior and vertical directions both show biphasic patterns [25, 27].

### NIRS measurement

**Fig. 2:**
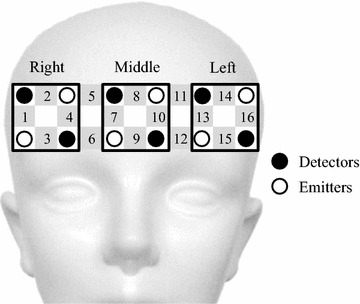
Schematic representation of near-infrared spectroscopy (*NIRS*) probes and channels. The NIRS system used in this study consists of six emitters (*white circles*) and six detectors (*black circles*), resulting in 16 source-detector pairs, called channels (*gray squares* with channel numbers). The distance between the source and detector probe in each channel is set at 3.0 cm. Signals from the four channels over the* right* prefrontal cortex (*PFC*) (No. 1–4),* middle* PFC (No. 7–10), and* left* PFC (No. 13–16), respectively, are averaged

We used a wearable 16-channel NIRS system (WOT, Hitachi Corporation, Japan) to evaluate activation in the prefrontal area when participants performed the smartphone task while walking. A portable processing unit for controlling the optical topography measurements was connected to the probe unit through a flexible cable bundle. The processing unit sent data to a personal computer that controlled the experiment through a wireless local area network. This system imposed no restrictions on movement due to wiring, and allowed subjects to walk freely within the area covered by the wireless local area network. Figure 2 shows a schematic of the NIRS probes and channels. The NIRS system used in this study consisted of six emitters and six detectors, resulting in sixteen channels, each consisting of one source-detector pair. The distance between source and detector probes in a channel was set to 3.0 cm. The lowest probes were positioned along the Fp1–Fp2 line according to the international 10–20 system used in electroencephalography. Changes in the concentrations of oxygenated (oxy) and deoxygenated (deoxy) hemoglobin (Hb) were calculated by using the absorbance change of 705- and 830-nm light according to the modified Beer–Lambert law [28, 29]. We used changes in oxy-Hb values as indicators of changes in regional cerebral blood volume, because oxy-Hb is more sensitive than deoxy-Hb as a parameter for measuring blood flow changes associated with brain activation [30]. The start of a session was manually marked on the NIRS data in response to an alerting sound produced by the iPod.

### NIRS Data analysis

The sampling frequency for the NIRS data was 5 Hz. We defined an analysis block as the period from 20 s prior to smartphone task onset in an experimental block as defined above to 10 s after smartphone task completion. Each participant’s data consisted of five blocks. Artifacts were detected as rapid changes in oxy-Hb concentration more than three standard deviations over the average for two consecutive samples [31]. All blocks that had been affected by motion artifacts were removed. Participants who displayed such motion artifacts in two blocks or more were excluded. A moving-average filter with a time window of 5 s was applied. A band pass filter of low pass 0.5 Hz was applied to account for the effects of Mayer waves and high-frequency fluctuations, and high pass 0.01 Hz was used to account for baseline drift. After deleting blocks containing artifacts, the data from the remaining analysis blocks for each participant were averaged.

A drawback of the NIRS method is the variability of the path length, which is dependent on the superficial scalp and tissue structure over the brain [32, 33]. To avoid these problems, the oxy-Hb data from each channel of each participant were normalized by a linear transformation so that the mean ± standard deviation of the oxy-Hb levels in the 10–20 s prior to the smartphone task condition were 0 ± 1 (AU). This normalization was also useful for circumventing the influence of differential path-length factors between participants and between cortical regions [34]. The NIRS data during dual task was defined for statistical analysis as the mean of the data recorded during a 30-s period of smartphone use while walking. To offset the low spatial resolution of NIRS and inter-individual anatomical variability, the four channels over the right PFC, over the middle PFC, and over the left PFC were averaged, respectively (Fig. 2). We used the Platform for Optical Topography Analysis Tools (Hitachi Corporation, Japan) and MATLAB software to analyze the NIRS data.

### Statistical analysis

We defined a dual-task cost on the step time and on the number of mistakes according to the following equation:

$${\text{Dual-task cost}}{{ = \left( {{\text{dual-task value}} - {\text{single-task value}}} \right)} \mathord{\left/ {\vphantom {{ = \left( {{\text{dual-task value}} - {\text{single-task value}}} \right)} {{\text{single-task value}} \times 100}}} \right. \kern-0pt} {{\text{single-task value}} \times 100}}.$$

We defined a dual-task cost on acceleration magnitude and number of correct responses according to the same equation but of opposite sign:

$${\text{Dual-task cost}}{{ = \left( {{\text{single-task value}} - {\text{dual-task value}}} \right)} \mathord{\left/ {\vphantom {{ = \left( {{\text{single-task value}} - {\text{dual-task value}}} \right)} {{\text{single-task value}} \times 100}}} \right. \kern-0pt} {{\text{single-task value}} \times 100}}.$$

In general, long step times, many mistakes, low acceleration magnitudes, and low correct-response numbers indicate worse performance. Therefore, higher values of these measures indicate larger dual-task costs for all parameters.

Clinical data, dual-task cost, and numbers of NIRS blocks rejected for artifacts were compared between the two age groups using Student’s *t* test or the χ2 test, depending on the type of variable assessed. A two-way repeated-measures analysis of variance (ANOVA) was used to determine the effects on gait and task parameters of age (young and older) as a between-participants factor and Condition (single and dual) as a within-participants factor. A two-way repeated-measures ANOVA was used to determine the effects on the NIRS data of Age as a between-participants factor, and of site (right PFC, middle PFC, and left PFC) as a within-participants factor. A post hoc analysis was performed using Bonferroni’s correction to reduce the possibility of Type I errors. To evaluate the correlation of PFC activation with cognitive and gait changes occurring during smartphone use while walking, each dual-task cost parameter (step time, acceleration magnitude, correct rate, and mistake rate) served as a dependent variable in a multiple regression analysis, with the NIRS data (right PFC, middle PFC, and left PFC) as an independent variable. Stepwise inclusion/exclusion of independent variables into the regression model was determined by *F* probability of *p* < 0.05 for inclusion and *p* > 0.1 for exclusion. Multiple regression analysis was performed separately for young and older groups, because of our finding of a significant difference in dual-task costs between these groups, as described below.

## Results

### Clinical data

Participants did not report any adverse side effects during the course of the study. No difference was observed between young and older adults in terms of sex ratio, but the MMSE scores of the young group (29.6 ± 0.8, range 27–30) were higher than those of the older group (28.3 ± 1.8, range 26–30) (*p* = 0.013). Height in the young group was higher than the older group in males (172.8 ± 5.6 vs. 166.1 ± 3.7 cm, *p* = 0.005) but not in females (162.4 ± 4.5 vs. 153.0 ± 9.8 cm). We excluded the data from one young participant (three artifact blocks) and one older participant (three artifact blocks) from the analysis due to artifacts. No significant difference in the number of NIRS blocks rejected for artifacts was found between young and older groups after excluding these two subjects (0.7 ± 0.5 blocks vs. 0.8 ± 0.4 blocks).

### Cognitive parameters

A two-way repeated-measures ANOVA for correct rate showed a significant effect of Age (*F*_1,27_ = 131.809, *p* < 0.001) and Condition (*F*_1,27_ = 9.885, *p* = 0.004), but no significant interaction effect between age and condition. Post-hoc testing revealed that the correct rate in the young group was larger than in older adults in both the single- (*p* < 0.001) and the dual-task conditions (*p* < 0.001). The correct rate in the dual-task condition was significantly lower than in the single-task condition in the older group (3.48 ± 0.89 vs. 4.50 ± 1.33; *p* = 0.018) but not in the young group (11.36 ± 2.25 vs. 12.12 ± 2.74).

A two-way repeated-measures ANOVA for mistake rate showed a significant effect of condition (*F*_1,27_ = 8.364, *p* = 0.007) and an interaction between age and condition (*F*_1,27_ = 5.037, *p* = 0.033), but no significant effect of age. Post-hoc testing revealed that the mistake rate increased significantly in the dual-task condition relative to the single-task condition in the older group (1.25 ± 0.71 vs. 0.90 ± 0.41; *p* = 0.001), but not in the young group (0.99 ± 0.52 vs. 0.94 ± 0.41).

**Fig. 3:**
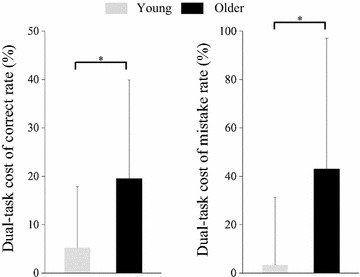
Dual-task cost of cognitive parameters. *p < 0.05, *error bar* standard deviation

Figure 3 shows the dual-task cost of cognitive parameters. The dual-task costs on correct and mistake rates in the older group were higher than in the young group (correct, *p* = 0.029; mistake, *p* = 0.018).

### Gait parameters

A two-way repeated-measures ANOVA for step time showed significant effects of condition (*F*_1,27_ = 19.816, *p* < 0.001) and of interaction between age and condition (*F*_1,27_ = 13.737, *p* = 0.001), but no significant effect of age. Post-hoc testing revealed that step time in the dual-task condition was significantly higher than in the single-task condition in the older group (537.49 ± 50.93 vs. 522.01 ± 50.73 ms; *p* < 0.001), but not in the young group (529.58 ± 34.81 vs. 528.17 ± 37.21 ms).

A two-way repeated-measures ANOVA for acceleration magnitude showed significant effects of Age (*F*_1,27_ = 5.034, *p* = 0.033), Condition (*F*_1,27_ = 94.396, *p* < 0.001), and an interaction between Age and Condition (*F*_1,27_ = 6.738, *p* = 0.015). Post-hoc testing revealed that acceleration magnitude in the young group was larger than in the older group in the dual condition (*p* = 0.014) but not in the single condition. Acceleration magnitude in the dual condition was less than in the single condition in both young (1.93 ± 0.55 vs. 2.07 ± 0.56 m/s^2^; *p* < 0.001) and older groups (1.51 ± 0.25 vs. 1.75 ± 0.32 m/s^2^; *p* < 0.001).

**Fig. 4:**
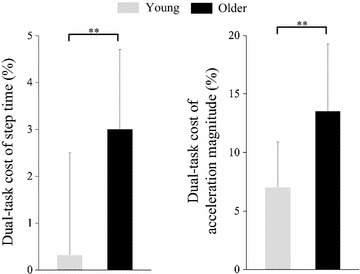
Dual-task cost of gait parameters. **p < 0.01, *error bar* standard deviation

Figure 4 shows the dual-task cost of gait parameters. Dual-task costs for step time and acceleration magnitude were larger in the older than in the young group (step time, *p* = 0.001; acceleration magnitude, *p* = 0.001).

### NIRS data

**Fig. 5:**
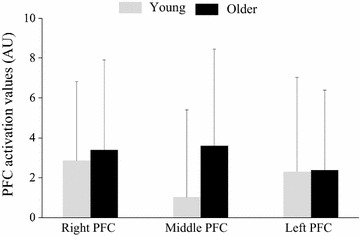
NIRS data. *Error bar* standard deviation, *PFC* prefrontal cortex

Figure 5 shows the NIRS data for the dual task in both groups. A two-way repeated-measures ANOVA for NIRS values showed no significant effect of Age or Site, nor were there any statistically significant interactions.

### Correlation of PFC activation with dual-task cost

**Fig. 6:**
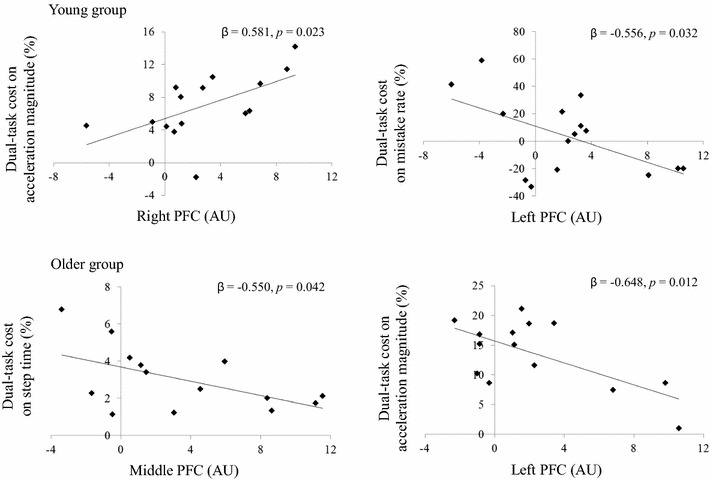
Correlation of PFC activation with dual-task cost. *PFC* prefrontal cortex

Figure 6 shows the correlations between PFC activation and dual-task cost. In young adults, multiple regression analysis revealed an association between right PFC and dual-task cost on acceleration magnitude (*R*^2^ = 0.338, *F* = 6.629, *β* = 0.581, *p* = 0.023) and between left PFC and dual-task cost on mistake rate (*R*^2^ = 0.309, *F* = 5.803, *β* = −0.556, *p* = 0.032). In older adults, multiple regression analysis revealed a negative association between middle PFC and dual-task cost on step time (*R*^2^ = 0.302, *F* = 5.199, *β* = −0.550, *p* = 0.042) and between left PFC and dual-task cost on acceleration magnitude (*R*^2^ = 0.419, *F* = 8.668, *β* = −0.648, *p* = 0.012).

## Discussion

The purpose of this study was to determine the extent to which PFC activity, as measured by the NIRS, reflects the cognitive and physical demands of smartphone use during walking. In the present study, the degree of PFC activation during smartphone use while walking did not differ between young and older adults. However, our results suggest that the influence of PFC activation on cognitive and physical dual-task costs differed across age groups. In young adults, the left PFC inhibited inappropriate action and the right PFC stabilized gait performance during smartphone use while walking. In older adults, a less-lateralized PFC activity pattern suppressed the deterioration of gait performance, but this PFC activation resulted in impairment of the cognitive task. These results suggest that lateralization of motor and cognitive tasks aids in efficient task completion during a complex action such as using a smartphone while walking.

### Dual-task cost differs across age groups

Our study showed that cognitive task performance during smartphone use while walking was impaired in older not but young adults. Smartphone task prolonged step time in the older group and decreased acceleration magnitude in both age groups. In addition to basic differences in physical and cognitive abilities, these findings might result from lower visual ability and/or unfamiliarity with smartphone use in older adults. Moreover, we found that the dual-task costs for physical and cognitive performance were more severe in the older group than in the young-adult group. There is clear evidence of dual-task cost, indicating that cognitive-motor interference can cause deterioration of one or both tasks [5, 6]. A far more consistent pattern of results can be found in older adults, where it is commonly reported that there is a reduction in walking speed and cognitive performance under dual-task walking. In line with these data, our study showed that smartphone use while walking prolonged step time and reduced acceleration magnitude while impairing cognitive performance in older subjects. These results are consistent with previous reports showing that gait stability was prioritized in older adults with low postural control ability during dual-task walking [5, 10]. In addition to the effect of postural control ability, cognitive function, in particular, attention and executive function, has been linked to dual-task cost [5, 9]. It is generally considered that overall cognitive function decreases with aging, especially executive functions such as monitoring and attention [17, 18]. Several studies have reported that lower measures of executive function are associated with increasing deterioration of gait performance during dual-task walking [9, 35, 36]. Therefore, it is likely that our observed increase in dual-task cost in older adults resulted from the decline of executive function in this group.

On the other hand, young adults could play a smartphone game while walking without deterioration of cognitive performance, although they reduced their magnitude of acceleration. These results indicate that the young adults allocated more attention to the cognitive task compared with older adults, but that they could also pay attention to gait stability. Recent work has proposed that cognitive tasks may be prioritized depending on postural control ability, self-awareness, and task complexity [8]. In this model, healthy individuals who have sufficient postural control can elect to prioritize a cognitive task over gait stability during dual tasks. Our data from smartphone use while walking partly support this hypothesis; however, young adults could appropriately allocate attention to two simultaneously performed tasks.

### PFC activity reflects cognitive and physical demands in smartphone use while walking

Previous studies have shown that a dual task increased oxy-Hb levels in PFC regions relative to a single task, but it remains controversial whether the PFC is more strongly activated in young or older adults during the performance of dual tasks [13, 19]. In the present study, time series measurements of oxy-Hb values during dual task performance were not significantly different between young and older adults. This discrepancy may be related to differences between previous studies and the present study in task design and methods of analysis of the NIRS data. On the other hand, we found that the influence of PFC activation on dual-task cost during smartphone use while walking differed across age groups. In young adults, left PFC activation reduced dual-task cost on the rate of mistake responses, and right PFC activation increased dual-task cost on acceleration magnitude during smartphone use while walking. These results suggest that left PFC activity may inhibit inappropriate action and right PFC activity be involved in a more conservative basic gait pattern for gait stability involving a reduced magnitude of acceleration. A previous study suggested that sufficient postural control ability and self-awareness allow healthy participants initially to allocate more attention toward the cognitive task than toward gait stability [8]. However, considering the correlation between PFC activation and dual-task cost, young adults might divide their attention between the cognitive task and gait stability equally.

In contrast, PFC activation in older adults correlated only with dual-task cost on gait, without effects on cognitive performance. In aging states, deterioration of postural control may cause alterations in balance and postural responses [37]. As a result, it becomes difficult to devote the same attention to cognitive performance. In fact, our results showed that older adults exhibited the more conservative gait pattern, reducing their walking speed and acceleration magnitude that was concomitant with impaired cognitive performance during smartphone use while walking. Therefore, we predicted that PFC activation would induce a conservative basic gait pattern as we had observed in young adults. However, in contrast to our expectation, PFC activation negatively correlated with dual-task cost on gait performance in healthy older adults. These results indicated that PFC activation might suppress the dual-task cost on gait performance to prioritize physical demand during smartphone use while walking. Of course, older adults might have felt difficulty in reducing walking speed and acceleration magnitude during smartphone game playing while walking, because they were less familiar with smartphones than young adults were. However, these differences in the correlation of PFC activity with gait function between young and older adults might also be explained from the monitoring system point of view. A previous paper suggests that the PFC contributes to monitoring of self-performance [38]. Therefore, the PFC might have to strongly activate to monitor gait performance and properly judge risk when dual-task cost is small in older adults. This may represent a compensatory mechanism by the PFC to ensure gait stability. As a result, the PFC in our older group had no capacity to cope with cognitive function, unlike in the young adults. In summary, the right PFC in young adults induced a dual-task cost that compensated for gait stability, but the left and middle PFCs in older adults suppressed the dual-task cost on gait performance and/or monitored gait instability during smartphone game playing while walking.

As described above, the roles of the individual sides of PFC in cognitive and physical tasks were clearly different in young adults. These results might be consistent with prior findings of PFC lateralization in executive function [39–41]. In general, lateralization is thought to allow each hemisphere to process information without interference by the contralateral hemisphere [42, 43]. Several studies have suggested that the speed of transcallosal conduction is limited in larger brains, which implies that the transfer and integration of information between the hemispheres through the corpus callosum require more time and energy in humans [44, 45]. Therefore, it may be more efficient for smartphone use while walking that each hemisphere works independently. In contrast, the hemispheric asymmetry reduction in older adults (HAROLD) model has been generally proposed as a theory of neural compensation for declining cognitive function during aging [46]. The HAROLD model posits that as age reduces the capacity for neuronal processing in each hemisphere, the hemispheres are required to work together bilaterally to solve a given task. In the present study, the dual-task cost of gait performance in older adults correlated with left and middle PFC activation, but not with right PFC activation. Thus, these activation patterns are not a typical HAROLD phenomenon, but are consistent with this concept, as the PFC might be required to activate more widely to focus on gait performance during smartphone use while walking in older adults.

### Limitations and future studies

Unlike in young adults, PFC activity in healthy older adults was unable to sufficiently prolong step time and reduce acceleration magnitude to prioritize gait stability. Although this difference might result from less habituation to smartphone use as discussed above, this failure to enforce a posture-first strategy by the PFC might contribute to the fundamental factor that some older adults and patients with neurological disease inappropriately use a posture-second strategy [8, 47]. In this model, individuals are unable to properly judge the risk of their actions and inadvertently exacerbate their fall risk in dual-task situations. Therefore, elucidating the association between PFC activation and dual-task walking among patients with neurological diseases such as stroke and Parkinson’s disease may reveal the mechanism of posture-second strategy and fall risk. There is growing evidence that dual-task training improves executive function and the ability to divide attention more effectively than either physical or cognitive training alone [48, 49]. Further study is required to elucidate a more detailed mechanism of prioritization, to reveal whether change in PFC activity after dual-task training will influence dual-task cost. Furthermore, dual-task training might lead to better dual tasking by ameliorating the reduced lateralization of PFC activity seen in older adults.

There are several limitations of this study to be considered when interpreting these results. First, it is important to address potential confounds related to cognitive-motor interference and NIRS activation resulting from our design using a smartphone. In order to exclude the possibility that the gait pattern was altered because of decreased availability of visual information about the walker’s surroundings rather than because of increased cognitive demands, participants were instructed to keep their faces turned the smartphone screen while both walking alone and dual-task walking. However, this instruction itself might give priority to the cognitive over the physical demand. Therefore, to investigate a more precise mechanism for prioritization during smartphone use while walking, we should compare the data under dual-task instructions such as “focus on smartphone use,” “focus on walking,” and “focus on either task over the other by free decision.” Dual tasks using other cognitive tasks, such as verbal fluency or calculation, should also be used to clarify the cognitive and physical prioritization in the absence of visual instruction. Additionally, the lack of adjustment of the smartphone task difficulty for each subject may have caused the high level of variability in NIRS data seen here; this may have impaired our ability to uncover differences in NIRS data with regard to age and site.

Another limitation of the interpretation of these results is issues with evaluation parameters. This study did not assess executive function directly. We evaluated step time and acceleration magnitude as our gait measures, not but gait stability *per se*. Furthermore, we evaluated only the PFC activity using the wearable NIRS. Future studies will be needed for a more detailed evaluation of gait parameters and activity in other brain regions using multi-channel NIRS to understand the mechanism of prioritization between cognitive and physical demands during smartphone use while walking. Moreover, we did not directly compare NIRS data of smartphone use while walking with that of single tasks such as smartphone use or walking alone. In the future study, we should set sufficient intervals of walking alone between dual-task conditions for excluding pre- and post-activation factors in the analysis of the single-task condition. For precisely comparing NIRS data between single-task and dual-task conditions, it also might be desirable to design the study protocol to treat each condition (smartphone use alone, walking alone, and dual task) as a task and standing as a control.

Lastly, aging-related changes in functional hemodynamics might not be associated with changes in neural processing *per se*, but could rather be a consequence of neurodegeneration and cortical atrophy with aging, affecting NIRS sensitivity. The path length of near infrared light and the NIRS sensitivity are dependent on the scalp-to-cortex distance [32, 33]. To circumvent these issues, the NIRS data from each channel of each participant were normalized by linear transformation. However, future studies should address the impact of anatomical differences due to cortical atrophy, frontal sinus and skull thickness by using imaging [32, 33]. Moreover, it is necessary to monitor extra cortical physiological response such as blood pressure, heart rate and skin blood flow, which influence the NIRS measurements [50, 51].

## Conclusions

The influence of PFC activation on dual-task cost during smartphone use while walking differed across age groups. In young adults, the left PFC inhibited inappropriate action and the right PFC stabilized walking performance during dual tasks. These results may provide support for the brain lateralization theory. However, PFC activity in older adults was less lateralized for suppressing dual-task cost on gait performance during dual-task walking, resulting in inability to cope with a cognitive demand. It may be more efficient for appropriately allocating attention to simultaneously performed cognitive and physical tasks if each hemisphere works independently on each task.

## Abbreviations

PFC: prefrontal cortex
NIRS: near-infrared spectroscopy
MMSE: Mini-Mental State Examination
ANOVA: analysis of variance
HAROLD: hemispheric asymmetry reduction in older adults
